# Atypical size and location of a right atrial myxoma: a case report

**DOI:** 10.1186/1752-1947-6-26

**Published:** 2012-01-23

**Authors:** Vinícius JS Nina, Nathalia AC Silva, Shirlyne FD Gaspar, Thaísa L Rapôso, Eduardo C Ferreira, Rachel VAH Nina, Joyce S Lages, Fernando ACC Silva, Natalino Salgado Filho

**Affiliations:** 1 University Hospital of the Federal University of Maranhão, Rua Barão de Itapary, 227 Centro 65020-070, São Luis Maranhão, Brazil

## Abstract

**Introduction:**

Primary intracardiac tumors are rare and approximately 50% are myxomas. The majority of myxomas are located in the left atrium and have variable clinical presentation. We report a case of a large myxoma in the right atrium, which is an uncommon location for this type of tumor.

**Case presentation:**

A 45-year-old Caucasian woman with a history of palpitation had dyspnea on great exertion and discrete weight loss. A cardiac evaluation showed splitting of S1. An echocardiogram showed a large mass in the right atrium, suggesting myxoma; chest computed tomography confirmed the diagnostic hypothesis. Our patient underwent surgical treatment with excision of a 10 cm multilobulated mass. She presented with supraventricular tachycardia during the operation. She was placed in the intensive care unit and her condition improved after the use of amiodarone. The diagnosis of myxoma was confirmed by histopathological study.

**Conclusions:**

In this case report, we emphasize the rarity of large myxomas in the right atrium and the difficulty of differential diagnosis given their dimension and location.

## Introduction

Cardiac tumors represent 0.2% of all tumors found in humans [1]. These tumors are divided into primary and secondary or metastatic. Secondary or metastatic tumors are 20 to 40 times more frequent than primary tumors. Primary intracardiac tumors are rare. Approximately 75% are benign, and approximately 50% are myxomas, which have an incidence of 0.0017% in the general population. Histologically, these are real tumors, derived from multipotent mesenchymal cells of the subendocardium [1-4].

Myxomas are located in the left atrium in 75% to 80% of cases and are almost always present with signs and symptoms of mitral valve disease or thromboembolic events. They may arise in other locations, such as the right atrium (RA) (18% of cases) [5], and, more rarely, in the aorta, pulmonary artery, ventricles, vena cava, or even other organs [3,6]. The differential diagnosis is performed mainly between rhabdomyoma or thrombus [2].

Myxomas affect patients within a wide age range (15 to 80 years), and the average age is approximately 50 years. There is a female predominance in the sporadic form [7]. Myxomas are usually pedunculated, solitary, and sporadic but may be associated with familial autosomal dominant syndromes (7% of cases) [6]. These familial forms have the following characteristics: they affect young male patients, they are multiple, and they have a risk of recurrence after surgical excision [4].

In regard to the macroscopic aspect, the surface of myxomas may have smooth or lobulated macroscopic features. Oval, rounded, and irregular shapes have been described, and a brownish color appears to be predominant. The consistency of myxomas is also variable, from firm to gelatinous.

Microscopic features of myxomas are characterized by a myxoid matrix rich in mucopolysaccharides, in which polygonal cells with an eosinophilic cytoplasm can be detected. The polygonal cells may appear as a star or nest shape or may be multinucleated. Microscopic characteristics including mitoses, necrosis, or pleomorphism are usually not detected or is eventually present as mild findings.

Although this tumor presents with benign histological features, the signs and symptoms caused by myxomas are atypical and highly variable and can result in a difficult diagnosis of this neoplasia [3,8]. According to the size, mobility, and location of the tumor as well as physical activity and body position, patients' symptoms may have an asymptomatic course or progress with thromboembolic events that may even lead to sudden death [3,4,9].

The classic triad found in patients with cardiac myxoma is characterized by obstruction of blood flow, constitutional symptoms, and thromboembolic events [3,4,7]. The obstruction of blood flow leads to intermittent heart failure, and, similar to systemic non-specific flu-like malaise symptoms, there is usually a low fever of long duration, arthralgia, anorexia, and thromboembolic events [7]. RA myxoma, in particular, can obstruct the tricuspid valve, causing signs and symptoms of right heart failure, peripheral edema, ascites, hepatic congestion, and syncope [7]. Cardiac auscultation in atrial myxomas may vary with the size, location, mobility, and prolapse of the tumor through the atrioventricular valves and even body position, and, therefore, detection of a murmur may or may not occur. An auscultation characteristic of myxoma is the 'tumor plop', which is an onomatopoeic representation of the heart sound caused by the presence of the tumor inside the atrial chamber that occurs in 15% of cases [7].

Routine laboratory assessment may show non-specific changes such as anemia, increased erythrocyte sedimentation rate, increased levels of globulin and C-reactive protein, leukocytosis, thrombocytopenia, and polycythemia [7]. Recent studies suggest that cardiac myxomas produce and release into the circulatory system an interleukin, which may be responsible for inflammatory or autoimmune phenomena.

Although transthoracic echocardiography is less invasive and presents an excellent sensitivity in detecting 95% of myxomas, the sensitivity increases to 100% when followed by transesophageal echocardiography [7]. Computed tomography (CT) and magnetic resonance imaging may be useful to demonstrate the point of fixation and associated complications. An electrocardiogram may be normal or show unspecific repolarization changes or even arrhythmias or heart block due to direct infiltration of cardiac conduction tissue or by irritating the myocardium itself or both. Chest X-rays and electrocardiograms are non-specific [2].

Once a cardiac myxoma is diagnosed, surgical excision should be performed without delays because of the constant risk of thromboembolic events [1,10]. Generally, surgical treatment is definitive and recurrence is uncommon. This report describes a rare clinical case of a large RA myxoma, highlighting the difficulty of the differential diagnosis of this tumor because of its unusual location and dimensions.

### Case presentation

A 45-year-old Caucasian woman had a 14-year history of palpitations, which became more frequent and intense in the last two months prior to admission and which were associated with dyspnea on exertion and weight loss of two pounds per month. She was on atenolol 50 mg/day and acetylsalicylic acid 200 mg/day.

**Figure 1:**
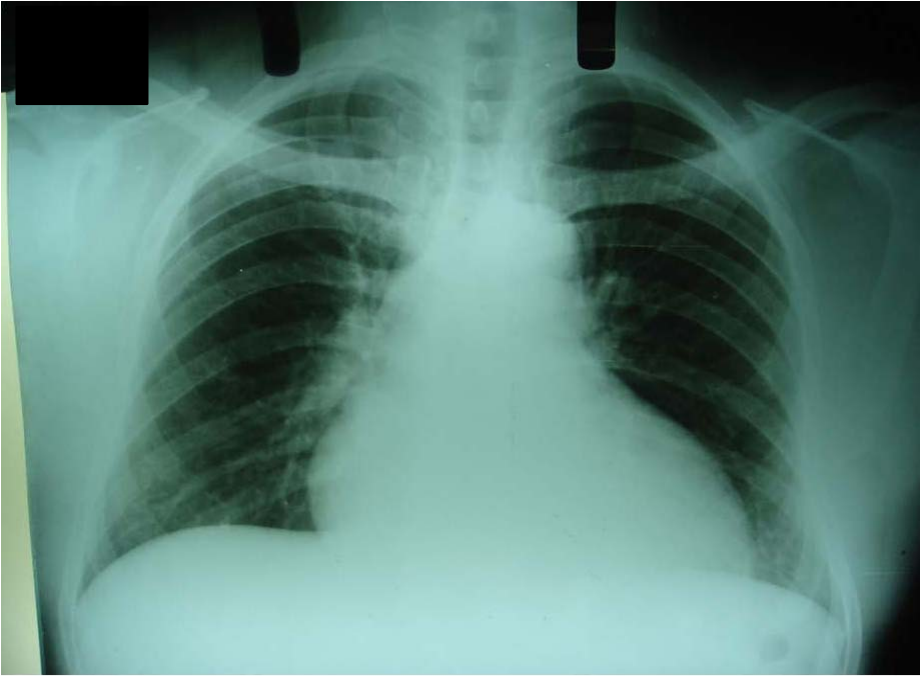
**A chest X-ray shows right atrial enlargement**.

**Figure 2:**
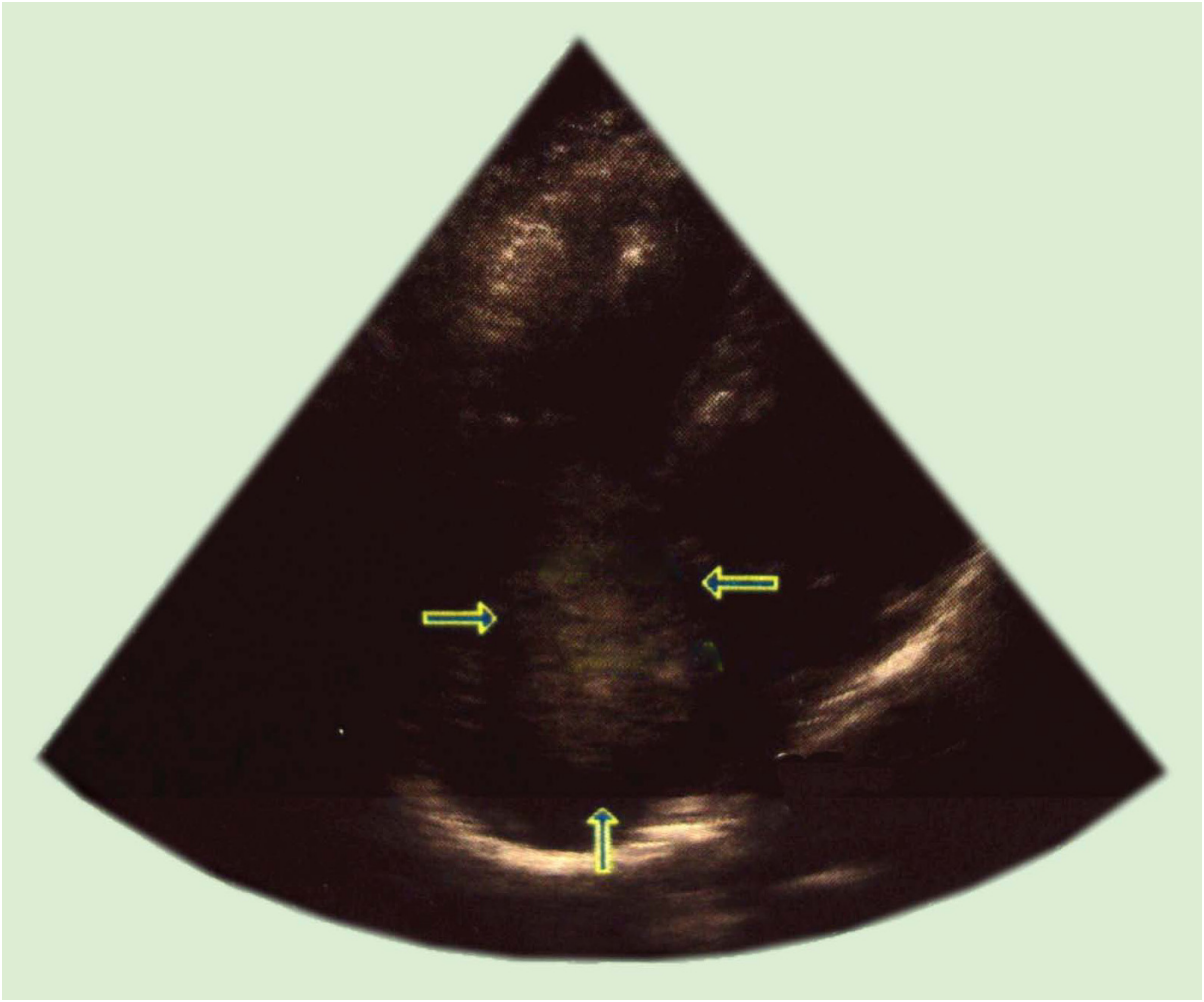
**A transthoracic echocardiogram shows a mass in the right atrium before an operation**.

Cardiovascular examination revealed that her heart rhythm was regular with splitting of S1 and no murmurs. The results of an examination of other systems were normal. A chest X-ray showed clear lung fields with RA enlargement (Figure 1). An electrocardiogram showed sinus rhythm with right bundle branch block and anterosuperior divisional block. A transthoracic echocardiogram showed a moving mass in the RA (Figure 2) attached to the atrial septum, an increased RA, right ventricular volume overload, and a dilated inferior vena cava.

**Figure 3:**
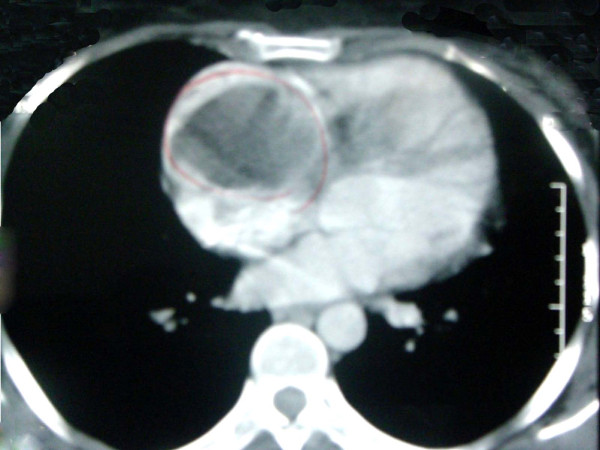
**An image of a chest computed tomography scan with intravenous contrast shows an enlarged cardiac silhouette with intra-atrial hypodensity**.

A total abdominal CT after oral and intravenous administration of iodinated contrast showed a normal-size liver and a heterogeneous enhancement of parenchyma associated with dilatation of the hepatic veins and inferior vena cava (cardiogenic liver). A chest CT scan after intravenous administration of iodinated contrast showed an enlarged cardiac silhouette with an intra-atrial hypodensity measuring approximately 6.1 × 5.6 cm and avoided filling of the RA with the contrast medium, suggesting the diagnosis of myxoma (Figure 3).

After providing written informed consent, our patient underwent median sternotomy under general anesthesia. Cardiopulmonary bypass (CPB) was established with conventional mild hypothermia (34.0°C). Cardiomegaly due to RA and right ventricular enlargement was observed. During an anoxic arrest for 17 minutes with single aortic cross-clamping, the tumor was completely excised through a right longitudinal atriotomy. The tumor was mobile, pedunculated, lobulated, clear, and gelatinous, with implantation at the atrial superior vena cava junction. The resected mass was sent for histological assessment. CPB was discontinued without any problems and our patient remained hemodynamically stable. The procedure was concluded in the usual fashion. Our patient was transferred to the intensive care unit (ICU) in good hemodynamic condition.

Postoperative complications included supraventricular tachycardia, which was reversed with intravenous amiodarone. Subsequently, our patient developed bradycardia for which cardiac pacing was required in the VVI (ventricular-based) mode. Sinus rhythm was resumed three hours later. Our patient was discharged from the ICU on the second postoperative day and from the ward on the fifth postoperative day. She was readmitted to the hospital twice while complaining of palpitations. However, in both admissions, the results of blood tests and imaging exams were normal and there was no evidence of recurrence of the tumor.

**Figure 4:**
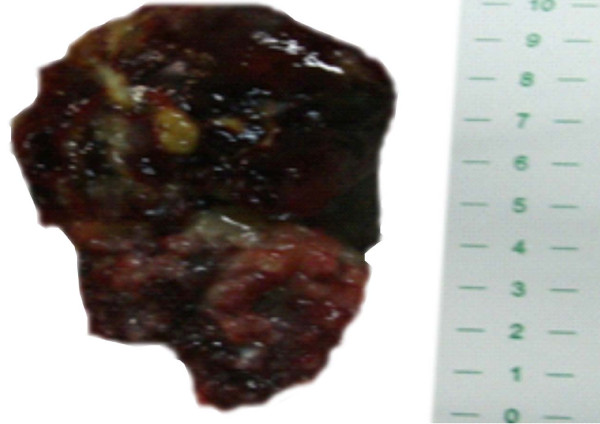
**A lobulated mass with an irregular surface, polypoid areas, and spots of loose and friable tissue**.

**Figure 5:**
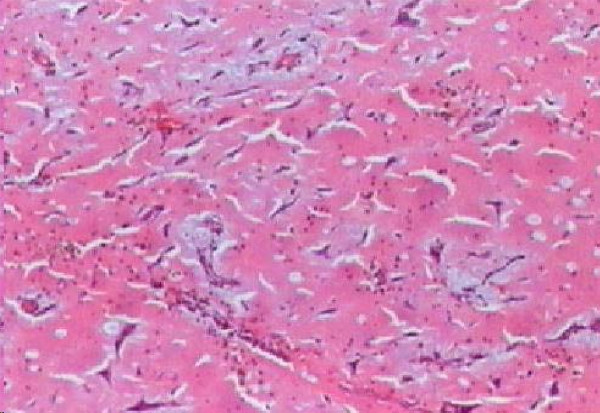
**An optical microscopic view of myxoid stroma with stellate cells without necrosis, mitotic activity, atypia, or pleomorphism**.

Macroscopically, the tumor presented as a 10 × 6 × 8 cm lobulated grayish mass weighing 100 g and had an irregular surface and polypoid areas of elastic consistency and spots of loose and friable tissue (Figure 4). On microscopy, there was abundant myxoid stroma with stellate cells. Necrosis, mitotic activity, atypia, and pleomorphism were not detected (Figure 5). A pathological examination confirmed the diagnosis of myxoma.

## Discussion

Primary cardiac neoplasms are rare and occur with an estimated incidence of 0.0017% to 0.19%, representing less than 5% of all heart tumors [11]. Myxoma is the most prevalent primary cardiac tumor. The RA is an unusual location and is the site of 15% to 20% of cases of myxoma [5]. A low incidence of RA myxoma has been reported for decades in several series of autopsy cases. Approximately 70% of affected patients are women [2,12] predominantly between the third and sixth decades of life [10,13], as was the case of the 45-year-old patient described in this report.

RA myxomas usually originate in the *fossa ovalis *or base of the interatrial septum [8], but in this case, the myxoma was implanted in the atrial superior vena cava junction. Myxomas are usually polypoid and pedunculated tumors (approximately 83% of cases) [10]. In this report, our patient had a 10 × 6 × 8 cm solitary, pedunculated mass with polypoid areas and a lobulated surface.

In a recent publication reporting 19 years of experience with surgical treatment of primary intracardiac myxoma, seven (17%) cases out of 41 originated from the RA. However, in this series, the mean maximal diameter of the tumors was 5.1 ± 1.8 cm [14]. To the best of our knowledge, our case is one of the largest RA myxomas described in the literature.

The signs and symptoms of RA myxomas are atypical and highly variable, depending on the size, position, and mobility of the tumor, and are modified according to physical activity and body position of the patient [7]. RA myxomas may remain asymptomatic [15] or eventually cause constitutional signs and symptoms, including fever, weight loss, arthralgias, Raynaud phenomenon, anemia, hypergammaglobulinemia, and an increased erythrocyte sedimentation rate due to the production of interleukin-6 [10]. These symptoms disappear after the tumor is removed [10]. In this report, our patient denied fever, arthralgias, and anemia but complained of weight loss (2 kg in two months) as the only constitutional sign.

Patients may also present with atypical chest pain, syncope, lethargy, malaise, palpitation, peripheral edema, pulmonary embolism, and hemoptysis. However, the most common manifestation is dyspnea (in 80% of patients), and right heart failure has been reported. Palpitations and dyspnea on exertion were found in this case. An abdominal CT scan showed hepatic congestion, a sign of right heart failure, but our patient had no ascites or edema in the lower extremities. Echocardiography remains the best diagnostic method for locating and assessing the extent of myxomas and for detecting their recurrence, with a sensitivity of up to 100%. However, transthoracic echocardiogram may not identify tumors smaller than 5 mm in diameter, and a transesophageal echocardiogram is required when there is suspicion of a very small tumor [12]. In this case, an echocardiogram suggested the hypothesis of RA myxoma, which was confirmed by a histopathological exam.

Although echocardiography is the modality of choice for screening cardiac masses, magnetic resonance imaging and CT provide information regarding tissue characteristics and allow an excellent overview of cardiac and paracardiac morphology. CT, in this case, showed an enlarged cardiac silhouette with an expansive ovoid mass in the RA with a density lower than that in the heart muscle [7].

The treatment of choice for myxomas is surgical removal [1,10]. Complete resection of the tumor and its implantation base with a good safety margin is essential to cure the disease, preventing recurrence and subsequent reoperations, which exposes the patient to other complications such as bleeding and the need for blood products. Myxomas are usually removed with a large resection of their pedicle or attachment to prevent recurrence. In this case, the tumor was located at the superior vena cava junction with the RA, making an excision with a large margin potentially dangerous because of the critical anatomical location and consequent high risk for conduction disturbance.

The recurrence rate of sporadic tumors is very low: between 1% and 3% [7]. The operative mortality ranges from 0% to 3% in multiple series [7]. The survival rate after surgery is elevated [10]. The surgical technique follows the basic concepts of cardiac surgery. However, some aspects should be taken into consideration in the surgical treatment of myxoma. Before resection, it is fundamentally important to clamp both the aorta and pulmonary trunk to avoid embolization of fragments because the myxomas are gelatinous and friable masses [8].

## Conclusions

Though a rare location for a large myxoma, the RA should always be considered in the differential diagnosis of a right-sided heart mass, especially when the patient shows signs and symptoms of heart failure with uncertain etiology. The findings in our case report suggest that cardiologists and surgeons need to make an early diagnosis and treat patients with these tumors to improve the prognosis.

## Abbreviations

CPB: cardiopulmonary bypass; CT: computed tomography; ICU: intensive care unit; RA: right atrium.

## Consent

Written informed consent was obtained from the patient for publication of this case report and any accompanying images. A copy of the written consent is available for review by the Editor-in-Chief of this journal.

## Competing interests

The authors declare that they have no competing interests.

## Authors' contributions

VJSN analyzed and interpreted the patient data regarding the cardiac mass and performed the final review of the manuscript. NACS helped to conduct the review of the literature and made a major contribution to the writing of the manuscript. SFDG helped to conduct the review of the literature and wrote the case report. TLR helped to conduct the review of the literature and wrote the introduction. ECF described the surgical aspects of the procedure. RVAHN analyzed and interpreted the patient imagining data. JSL described the macroscopic examination of the tumor. FACCS translated and reviewed the manuscript. NSF performed the histological examination. All authors read and approved the final manuscript.
